# A Pine Is a Pine and a Spruce Is a Spruce – The Effect of Tree Species and Stand Age on Epiphytic Lichen Communities

**DOI:** 10.1371/journal.pone.0147004

**Published:** 2016-01-22

**Authors:** Sofia Bäcklund, Mari Jönsson, Joachim Strengbom, Andreas Frisch, Göran Thor

**Affiliations:** 1 Department of Ecology, Swedish University of Agricultural Sciences, P.O. Box 7044, SE-750 07 Uppsala, Sweden; 2 The Swedish Species Information Centre, P.O. Box 7007, SE-750 07 Uppsala, Sweden; Chinese Academy of Forestry, CHINA

## Abstract

With an increasing demand for forest-based products, there is a growing interest in introducing fast-growing non-native tree species in forest management. Such introductions often have unknown consequences for native forest biodiversity. In this study, we examine epiphytic lichen species richness and species composition on the trunks of non-native *Pinus contorta* and compare these to the native *Pinus sylvestris* and *Picea abies* in managed boreal forests in northern Sweden across a chronosequence of age classes. Overall, we recorded a total of 66,209 lichen occurrences belonging to 57 species in the 96 studied forest stands. We found no difference in species richness of lichens between stands of *P*. *contorta* and *P*. *sylvestris*, but stands of *P*. *abies* had higher total species richness. However, species richness of lichens in stands of *P*. *abies* decreased with increasing stand age, while no such age effect was detected for *P*. *contorta* and *P*. *sylvestris*. Lichen species composition progressively diverged with increasing stand age, and in 30-year-old stands all three tree species showed species-specific assemblages. Epiphytic lichen assemblages in stands of 30-year-old *P*. *contorta* were influenced by greater basal area, canopy closure, and average diameter at breast height, *P*. *abies* stands by higher branch density and canopy closure, and stands of *P*. *sylvestris* by greater bark crevice depth. Differences in lichen species richness and composition were mainly explained by canopy closure and habitat availability, and the greater canopy closure in mature *P*. *abies* stands promoted the colonization and growth of calicioid lichen species. Our results indicate that the non-native *P*. *contorta* have similar species richness as the native *P*. *sylvestris*. The main difference in lichen species richness and composition is between *P*. *abies* and *Pinus* spp. in managed forests of boreal Sweden.

## Introduction

Intensive forestry is currently practiced worldwide [1], and an increasing demand for wood products (timber, pulp, biofuel, etc.) calls for further intensification and development of management practices [2]. Intensively managed forests typically lack the structural complexity of unmanaged forests, resulting in lower levels of biodiversity and the risk of reduced ecosystem functioning [3, 4]. Production can also increase by establishing non-native tree plantations, a practice that is continuously expanding around the world [5]. However, forestry as currently practiced is already recognized as a serious threat to biodiversity [6], and further intensification might worsen the situation [7]. Identifying ways to maintain, or even increase, production capacity without further jeopardizing biodiversity presents a major challenge. To develop such management schemes requires basic knowledge about how the transition from native to non-native tree species influences native biodiversity. However, such information is often unavailable [8, 9], which limits the ability to evaluate different management schemes and to develop more sustainable options.

Introducing non-native species can influence native biodiversity in many ways. In general, habitat modifications that provide a limiting resource or increase habitat complexity are likely to facilitate native species (e.g. [10]), while competition and modifications in habitat and ecosystem processes are examples of mechanisms that often displace or inhibit native species [11]. Not all taxa respond alike [12], but large changes can be expected when the new species is very different from the preceding vegetation, for example, when grasslands are planted with or invaded by coniferous trees [13, 8].

Boreal forests are extensively utilized in forestry, and translocations of various tree species are common [14, 9]. Boreal forests are characterized by low tree species richness compared to tropical or temperate regions, and in regions such as Fennoscandia the forests are dominated by a few coniferous species [15]. Because tree species richness is low, the major part of the biodiversity in boreal forests is found within other groups of organisms. Epiphytic lichens are a species-rich group in boreal forests and are an important part of the forest diversity [16]. Besides providing food and shelter for other organisms (e.g. forage for reindeers and snails, nesting material for birds, and shelter for small insects), lichens can influence nutrient cycling in the forest [16, 15].

Epiphytic lichen diversity is influenced by the tree species they inhabit and the environment surrounding that tree. Factors controlling epiphytic lichen diversity are related to the characteristics of both the forest stand (e.g. stem density, canopy cover, tree basal area) and the individual tree (e.g. branch density, substrate diversity, bark structure, bark pH, and bark stability) [17, 16]. Many of these factors can be correlated to tree age or stand age, and it is well established that old trees are more lichen species rich than young trees [18, 19, 20, 21]. The importance of forest stand age can be linked to habitat development and colonization processes. During stand development, both stand and tree structures will change and influence the conditions for epiphytic lichens [22]. For example, old trees and forests are likely to provide more complex and variable habitats due to coarse bark structure and variable canopy closure, and thereby host more species [23]. Tree age also reflects how long the tree has been available for colonization [22].

Light availability is important for lichen growth and vitality [24, 25]. On the tree trunk, light conditions are influenced by tree architecture [26, 22], and low light availability on the trunk can have a negative influence on stem-living lichens [19]. The stability of the substrate is also important [27, 19]. For example, the barks of some *Pinus* spp. are coarse and more stable at the base but smoother and easily exfoliated higher up in the tree [28], thus providing epiphytic lichens with different habitat conditions in different parts of the tree. A rapid growth rate of the tree might reduce the stability of the bark, which can be detrimental to a variety of epiphytic lichen species [19, 27, 29]. Furthermore, the acidity of the bark is important for the lichen species composition, and lichen communities on *Pinus* spp. and *Picea* spp. are tolerant of rather acidic conditions (pH 3–4) [30, 28].

Swedish forest management is mainly focused on two coniferous tree species, Norway spruce (*Picea abies*) and Scots pine (*Pinus sylvestris*), but since the second half of the 20^th^ century Lodgepole pine (*Pinus contorta*) has been introduced as a forestry tree. *Pinus contorta* is native to North America and has the potential for a 36% higher production than *P*. *sylvestris* regardless of site index [31]. It has been introduced on a large scale and now covers about 500,000 ha, or about 2.1%, of the productive forest land in Sweden [32]. Planting non-native *P*. *contorta* might provide a novel ecosystem for epiphytic lichens compared to native conifers, but the faster growth rate of *P*. *contorta* is likely to inhibit lichen diversity (e.g. [19]). It is also suggested that canopy closure will occur earlier in *P*. *contorta* stands compared to native trees of Sweden [31] leading to lower light availability that might constrain lichen diversity [24, 25]. Hence, the introduction of *P*. *contorta* might have a strong influence on native lichen diversity with a subsequent impact on forest biodiversity.

The aim of this study is to investigate lichen composition and diversity in relation to the planting of a non-native tree species. More specifically, we examine how epiphytic lichen species richness, species composition, and total coverage on trunks differ between the non-native *P*. *contorta* and the two native tree species *P*. *sylvestris* and *P*. *abies* in managed boreal forests across a chronosequence of age classes. We hypothesized that lichen species richness would increase with increasing stand age for all tree species, provided the greater habitat area of larger trees and longer time for colonization and establishment. We hypothesized that lichen species richness and composition would differ between stands of *P*. *contorta*, *P*. *sylvestris* and *P*. *abies* due to differences in tree characteristics (e.g., bark crevice depth and branch density) and stand structure (e.g., canopy closure and basal area). Forming a completely new habitat, we also expected lichen species richness to be lower on the non-native and fast-growing *P*. *contorta*.

## Material and Methods

### Study area

The study area is situated in the northern boreal zone of Sweden [33] and consists of tree stands within a 30 km radius of the town of Dorotea (64° 15’N, 16° 24’E) in Västerbotten County. The annual mean temperature of the area is +1°C and the mean monthly temperatures range from −13°C in January to +13°C in July. The length of the growing period is around 140 days and the mean annual precipitation is around 700 mm, which is typical for inland parts of northern Sweden (data from the Swedish Meteorological and Hydrological Institute for the period 1961–1990). The area is dominated by managed coniferous forests, predominately monocultures or mixed stands of *P*. *sylvestris* and *P*. *abies*. About 10% of the forested area in the region is planted with *P*. *contorta* [34]. Deciduous trees, mainly *Alnus incana*, *Betula pendula*, *B*. *pubescens*, *Populus tremula*, and *Salix* spp. are present in the study area, but mostly in small groups or as single trees. The mean altitude of the studied stands is 355 m above sea level (minimum 260 m and maximum 583 m), and the forests consist of successional stages covering the entire forestry rotation period of approximately 100 years. About 70% of the forests are owned by a single forest company, SCA, which enabled us to gain access to a stand database with almost total landscape coverage. On the land owned by SCA, about 71% of the stands are productive stands younger than 60 years of age, about 13% are older than 110 years, and about 0.9% of the land consists of mires and other low-productive land not used for forest production. Forest management over a rotation period typically include soil preparation, regeneration (e.g., planting), pre-commercial thinning, commercial thinnings and final harvest.

We sampled three age classes of productive forests– 15 ± 2 years, 30 ± 5 years, and 85 ± 5 years—forming a chronosequence. In the study area, about 6% of the stands are productive stands 13–17 years of age, 12.3% are 25–35 years, about 3.3% are mature 80–90 years. Most 15 and 30-year old stands had been subjected to pre-commercial thinning and the 85-year old stands had been subjected to commercial thinning. Stands suitable for inventory were selected according to the following criteria: dominated (>70%) by any of the three focal tree species (*P*. *abies*, *P*. *contorta*, or *P*. *sylvestris*), being established after clear-cutting, and being of predominantly flat topography. All stands were of dry-mesic-moist ground moisture type, and these corresponded to the vegetation types “*pine forests of the cowberry type*” or “*spruce forests of the bilberry type*” after Pålsson [35]. In these vegetation types, both *P*. *sylvestris* and *P*. *abies* occur naturally and *P*. *contorta* is regularly planted. For the 15 year and 30 year age classes, we randomly selected 12 stands from each age class. In the case of the 85-year-old stands, 11 stands were selected for 85-year-old *P*. *abies* and one stand for 85-year-old *Pinus contorta*. The 85-year old stand of *P*. *contorta* was the only stand of that age class within the study area, and it is one of the oldest stands of *P*. *contorta* in Sweden.

### Sampling

The inventory was conducted during snow-free periods during the years 2009–2011. For each forest stand, a transect along the longest possible line was chosen beforehand from stand maps. The average size of the study stands was 24.7 ha, and the average transect length was 750 m. Data sampling took place on four randomly selected points along each transect. At each point, the following stand data were measured: stem density (stems ha^−1^), basal tree area (m^2^ ha^−1^), and canopy cover (%, estimated visually by the same two persons as the percent sky that was covered by trees within a 10 m radius of the sampling point).

At each of the sampling points, the closest living focal tree was inventoried for lichens (i.e., four trees per stand), and we measured the tree diameter at breast height (cm; 130 cm above ground), tree height (m), and depth of bark crevices (mm; mean depth from the four cardinal directions at breast height) of the sample tree. We also counted the number of branches in each metre interval along the trunk (0–0.99 m, 1–1.99 m, etc.). A branch had to be at least 10 cm long and 0.8 cm thick to be included.

For lichen data, young trees (15 and 30 years old) were inventoried up to the point where the trunk diameter was ≤5 cm. In most cases, it was necessary to cut down the tree to facilitate the inventory. The 85-year-old trees were not cut down, and thus inventoried with the help of a ladder up to 5.5 m in height. The inventory was conducted using a double plot of 2 × 25 cm^2^ (5 cm × 5 cm width × height per plot; S1 Fig). For the first 2 metres from the ground, the plot interval was 25 cm (i.e., plots were located at 0 cm (base), 25 cm, 50 cm, etc.). Starting from 2 metres, the plot interval was extended to 50 cm (i.e., plots were at 250 cm, 300 cm, 350 cm, etc.). The plot was rotated clockwise by one cardinal direction (north, east, south, west) between each consecutive plot (S1 Fig). The starting cardinal direction for the sampling was shifted for each tree in a clockwise direction. In the 85-year-old stands, all plots were sampled in the same cardinal direction for each tree, with the cardinal direction shifted clockwise for each of the four sampled trees in each stand.

The abundance of individual lichen species was estimated by their occurrence frequency within the sampling plot by dividing the sampling plot into four subplots of 2.5 cm × 2.5 cm (S1 Fig). The presence of all lichen species (including crustose, foliose, and fruticose lichen forms) on the bark was counted once per subplot, i.e., the maximum abundance per plot was 4 for each species. Lichens were sometimes brought to the lab for later identification. In addition to abundance data of individual species, the total cover (%) for all lichen species combined was noted for each 5 cm × 5 cm plot. For this study, 96 forest stands, 384 trees, and 10,104 plots were inventoried. The study was carried out on private forest company land and the owner SCA provided all the permission necessary to conduct the study on these localities. No specific permissions were required for these locations and field activities, since they did not include any protected localities or endangered/protected species (i.e., ordinary managed forests were studied).

### Statistical analysis

#### Sample-based rarefaction

We used sample-based rarefaction curves [36] to compare lichen species richness in *P*. *abies*, *P*. *contorta*, and *P*. *sylvestris* stands within the same age class. The curves were produced with EstimateS version 9.0 and calculated with 100 random re-samplings without replacement. The variance estimate among randomizations was unconditional, meaning that a robust visual comparison of the curve-associated confidence intervals was possible at comparable levels of sampling effort [37]. Differences in species richness were considered non-significant (*p* > 0.05) if confidence intervals overlapped according to Colwell et al. [37].

#### Species richness models

We modeled tree-level species richness using the generalized linear mixed modeling (GLMM) framework with forest stand as the random factor, thus we let the coefficients vary by forest stand as a means of dealing with non-independence of the data. For species richness, we fitted Poisson models with logarithmic link functions (e.g., [38, 39]). Tree species (*P*. *contorta* was set as the reference because it was the non-native species), stand age, basal area, branch density, canopy cover, diameter at breast height, and bark crevice depth were used as explanatory variables. All two-way interactions between stand age and tree species, as well the structural explanatory variables (i.e., tree species, basal area, branch density, canopy cover, diameter at breast height, and bark crevice depth) were included in the full model. In all models, we accounted for varying sampling effort on different trees by including the number of tree plots surveyed as an offset variable. All explanatory variables were standardized to allow for comparisons of their respective effect size (regression estimates) [38, 40].

A set of sub-models including all possible combinations of the explanatory variables was generated [41]. We used model averaging to assess the relative strength of support for all biologically relevant models, as recommended when the Akaike weights (wi) of the ‘best models’ are less than 0.9 [42]. In cases where two or more models achieve similarly high levels of support, model averaging of the ‘top model set’ can provide a robust means of obtaining parameter estimates [41]. The Akaike weight of a model is essentially its probability compared to the probability of the other plausible models, and the sum of the Akaike weights for all plausible models is 1. Thus we used an information theoretic or ‘IT’ approach for model selection, and we calculated Akaike’s information criterion adjusted for small sample size (AICc) and Akaike weights in the R library *MuMIn* [43]. The differences (Δi) in the AICc for each sub-model were used to rank the models with Δi <4 used as the threshold for a model to be considered as having support [44]. The relative variable importance (RVI) was estimated on a scale of 0 to 1 by summing the AICc weights across all sub-models in which the variable occurred. Better models have larger AICc weights, and consequently variables that contribute more to model fit will have a higher RVI. The precision of the model-averaged parameter estimates account for model selection uncertainty, which is included in the estimated range of the confidence intervals.

The GLMMs were calculated in the statistical software R 3.0.1 [45] using the add-on library *lme4* [46]. The standardization of explanatory variables was done using the *arm* package [40], and the *MuMIn* package [43] was used for the multimodel inference.

#### Species composition

We used one-way analyses of similarity (ANOSIM) [47, 48] in the PAST software package version 2.12 [49] to investigate differences in lichen species composition on the different tree species and forest age classes. The analyses were based on a Bray–Curtis similarity matrix built on average abundance values of each species as averaged by plot numbers from four individual trees per stand [48]. Running the multivariate analysis on all species, or excluding species with few occurrences (<5), did not influence the results. Thus we present results including all observed species. In total, 96 stands and 57 species were included in the analysis. Correlation coefficients between five environmental variables and the nonmetric multidimensional scaling (NMDS) scores were calculated and presented as vectors from the origin in ordination plots. ANOSIM generates an R-statistic that gives a measure of how similar groups are. Values most commonly range from 0 to 1, and a large positive R close to one signifies large differences between groups, while a value close to zero indicates there is little difference between groups [49]. Levels of significance (*p*-values) of the differences between assemblages were obtained by a permutation procedure with 10,000 replicates on the similarity matrices [49]. The NMDS in the program PAST was also used to generate a visual configuration of the species composition patterns [49, 50]. NMDS is an ordination technique suitable for community data and does not have any assumptions of normality or linearity [51]. Similarity Percentage analysis (SIMPER) used the Bray-Curtis similarity measure (multiplied by 100) to evaluate which lichen species were responsible for the observed difference between groups of samples [47]. Species that consistently contributed significantly to the average dissimilarity between stand types were considered discriminating species, i.e., they were characteristic of specific stand types. The single 85-year old *P*. *contorta* stand was included as a reference stand in the stand age chronosequence data used in the GLMM and the rarefaction graph, but omitted from all other analyses. We show the data from the 85-year old *P*. *contorta* stand in the summary graphs (together with information on the sampling effort) to allow the comparison of the single stand to other groups.

## Results

There were some differences in stand structure between the non-native *P*. *contorta* and the two native tree species, and in general, *P*. *contorta* had larger diameters than *P*. *abies* and *P*. *sylvestris* (Table 1). The size of stands in the 85-year old age class was smaller than the size of stands in 15- and 30-year old stands for all tree species (Table 1).

**Table 1:** Average values (± SD) for stand data. Variables recorded in the survey of stands of *Picea abies*, *Pinus contorta*, and *Pinus sylvestris* in different age classes in northern Sweden. Because there was only one stand of 85-year-old *P*. *contorta*, no SD is shown for that stand.

Tree species	Stand age (years)	Stand size (ha)	Basal area (m^2^ ha^−1^)	Canopy cover (proportion)	Branch density (branches m^−1^)	Diameter at breast height (cm)	Bark crevice depth (mm)
*Picea abies*	15	28.9 (15.3)	5 (1.2)	0.26 (0.08)	9.5 (2.0)	5.8 (0.5)	0.14 (0.16)
	30	35.8 (27.1)	13 (3.9)	0.58 (0.14)	9.7 (3.7)	8.6 (0.8)	0.69 (0.28)
	85	8.9 (4.1)	20 (4.4)	0.66 (0.07)	8.2 (2.5)	20.0 (4.3)	3.28 (0.78)
*Pinus contorta*	15	26.9 (17.0)	11 (3.3)	0.57 (0.15)	9.2 (1.6)	8.2 (0.5)	0.18 (0.21)
	30	29.3 (17.5)	23 (2.5)	0.62 (0.08)	8.7 (1.2)	12.2 (1.0)	1.23 (0.54)
	85	8.4	30	0.27	4.3	30.9	4.25
*Pinus sylvestris*	15	31.3 (19.1)	8 (2.2)	0.36 (0.12)	7.9 (1.2)	7.2 (0.4)	1.23 (0.49)
	30	29.9 (27.8)	19 (6.8)	0.40 (0.14)	8.8 (1.0)	11.7 (1.4)	1.98 (0.73)
	85	7.1 (3.6)	25 (4.2)	0.38 (0.07)	3.6 (2.5)	22.5 (1.8)	4.88 (0.91)

### Lichen species richness

**Fig 1:**
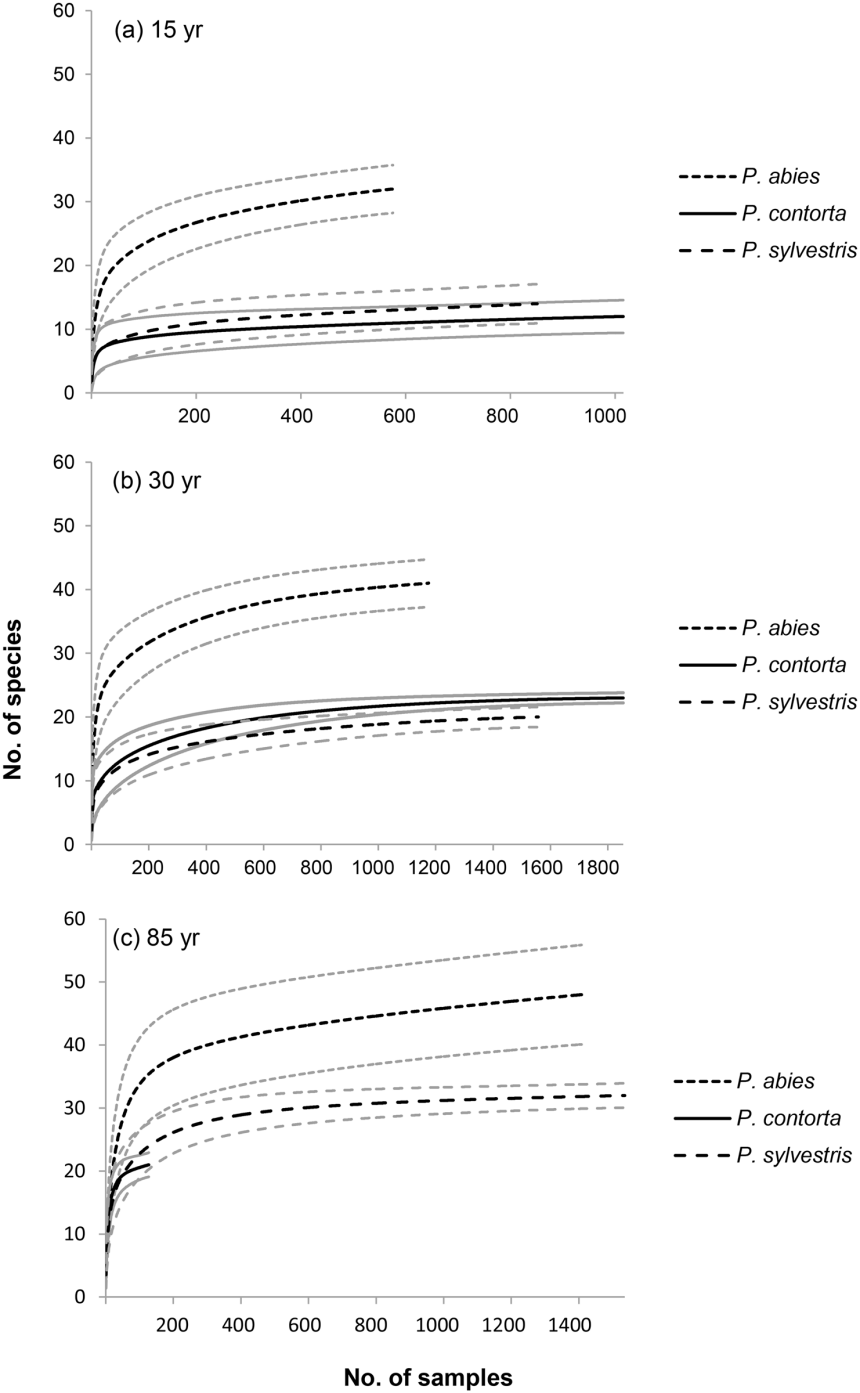
a-c. Sample-based rarefaction curves comparing plot-level species richness between *Picea abies*, *Pinus contorta*, and *Pinus sylvestris*. The three panels show (a) 15-year-old, (b) 30-year-old, and (c) 85-year-old stands in northern Sweden. A total of 12 stands and four trees were surveyed in each category, with the exception of the 85-year-old stands of *P*. *abies* and *P*. *contorta* where 11 stands and 1 stand, respectively, were surveyed. The light grey lines are 95% confidence limits.

We recorded a total of 66,209 lichen occurrences, of which 22% were crustose, 63% foliose, and 15% fruticose. In 28% of the subplots, no lichens at all were recorded. The recorded lichens belonged to 57 species. A total of 53 species were recorded for *P*. *abies*, 31 species for *P*. *contorta*, and 37 species for *P*. *sylvestris*. The sample-based rarefaction curves (Fig 1a–1c) all approached asymptotes (except for the 85-year-old stand of *P*. *contorta*), indicating that the sampling was sufficient to capture most of the lichen species present in the different stand types. The average number of species recorded per forest stand (all age classes pooled) was 23 species (range 9–32 species) for *P*. *abies*, 10 species (range 7–21 species) for *P*. *contorta*, and 12 species (range 6–24 species) for *P*. *sylvestris*. We found three near-threatened red-listed species, *Alectoria sarmentosa*, *Chaenotheca subroscida*, and *Chaenothecopsis nana* [52]. All occurrences of these three species were on *P*. *abies*. A complete list of all lichen species recorded is presented in S1 Table.

**Fig 2:**
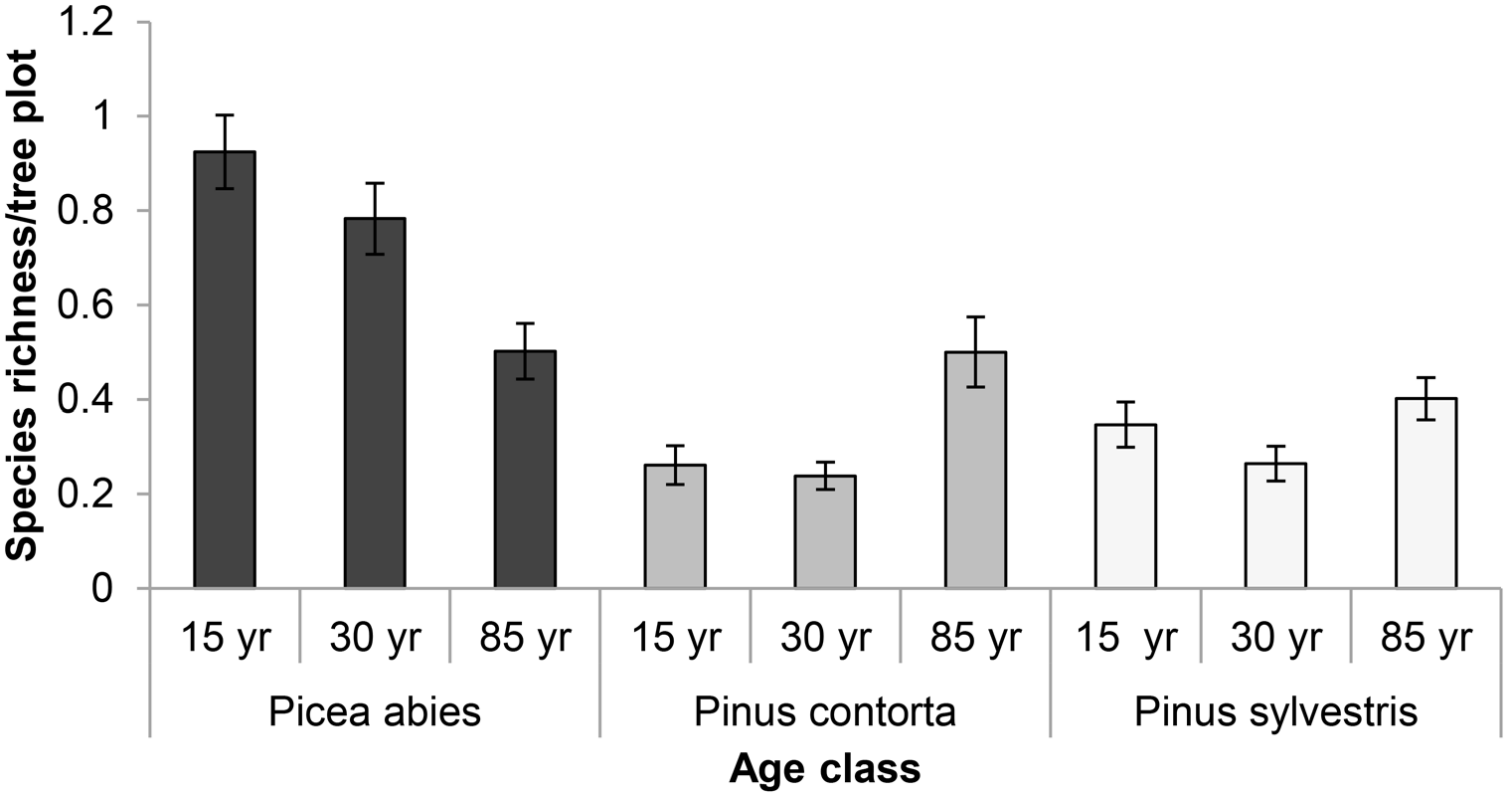
Average number of lichen species per tree plot in the different stand types. Error bars represent the standard error.

**Fig 3:**
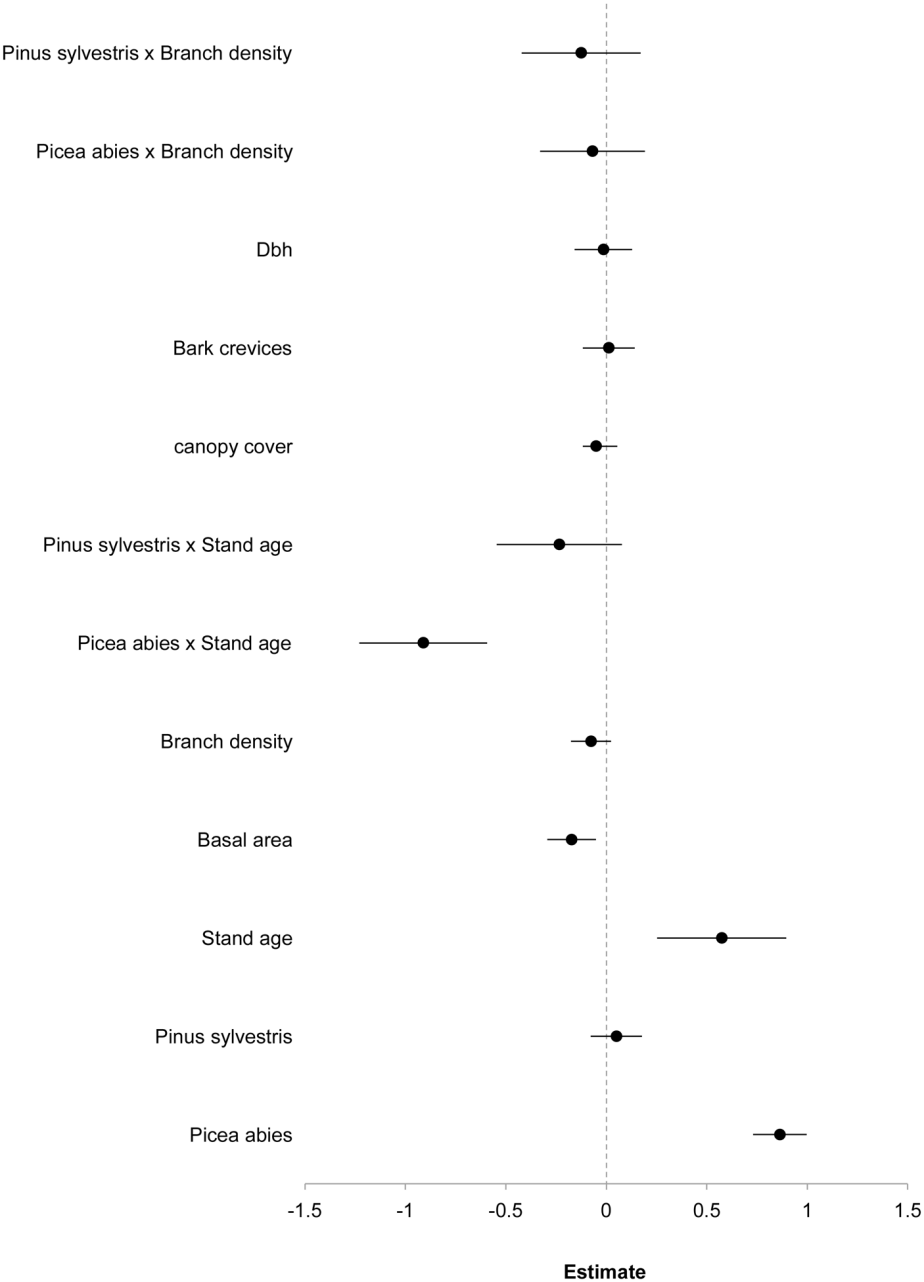
Model-averaged parameter estimates and 95% confidence intervals for total lichen species richness in *Picea abies*, *Pinus contorta*, and *Pinus sylvestris* stands of three different age classes.

**Fig 4:**
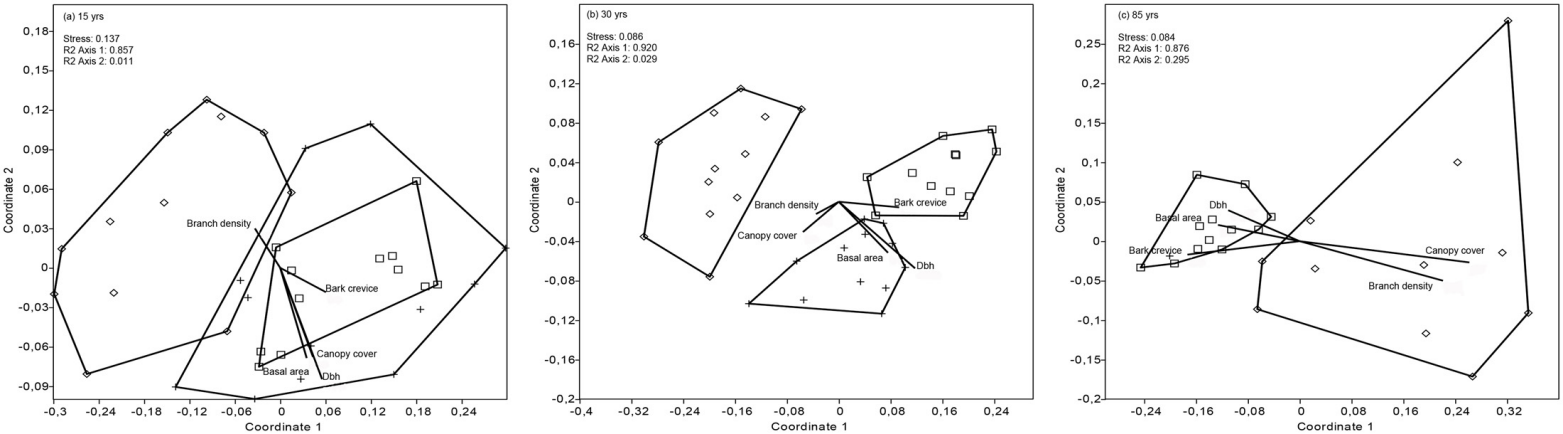
Nonmetric multidimensional scaling (NMDS) ordination graph of lichen species composition. Lichens of 57 taxa on 384 trees in a total of 96 managed forest stands of *Picea abies*, *Pinus contorta*, and *Pinus sylvestris* in (a) 15-year-old, (b) 30-year-old, and (c) 85-year-old stands. Triangles = *Picea abies*, crosses = *Pinus contorta*, and squares = *Pinus sylvestris*. Correlation coefficients between five environmental variables and the NMDS scores are presented as vectors from the origin. The lengths of the vectors are arbitrarily scaled to make a readable biplot, so only their directions and relative lengths should be considered. All NMDSs resulted in 2-dimensional solutions with all final stresses < 0.14. Axis 1 explained most of the variance in the data. The following terms have been abbreviated: bark crevice depth (Bark crevice) and diameter at breast height (Dbh).

Species richness differed between tree species, and total species richness was generally higher in *P*. *abies* stands than in both *P*. *contorta* and *P*. *sylvestris* (as indicated by the lack of overlap in 95% confidence intervals in Fig 1a–1c). After correcting for sampling effort (number of plots), species richness was still higher on *P*. *abies* (Figs 2–4). The coefficient of variation (CV) for species richness within stands ranged from almost no variation to about 50% (S2 Fig). Between stands, the coefficient of variation was slightly higher in young stands for all tree species (S2 Fig).

In accordance with our expectations, species richness was influenced by stand age. The GLMM results (corrected for the number of plots sampled) showed that besides tree species, stand age was the variable that best explained species richness in terms of both effect size and RVI (Fig 3 and S3 Fig). Tree species interacted strongly with stand age, and in stands of *P*. *abies* species richness decreased with increasing stand age. Despite the observation that total species richness was generally higher in older than younger stands, there was no effect of stand-age for *P*. *contorta* or *P*. *sylvestris* in the model (Fig 3 and S3 Fig). Species richness was negatively associated with basal area, which also had high RVI (Fig 3 and S3 Fig). Branch density had high RVI (S3 Fig) but a weak negative effect on species richness (Fig 3). Canopy cover showed only a weak effect and bark crevices and diameter at breast height had no effect, and all three of these variables had very low RVI (S3 Fig). Model statistics from the GLMMs are included in the supplementary S1 Model.

### Lichen species composition

The NMDS showed that species composition of *P*. *abies* stands deviated from those of *P*. *contorta* and *P*. *sylvestris* in all age classes (Fig 4a–4c). Species composition of *P*. *contorta* and *P*. *sylvestris* stands were similar in 15-year-old stands, but differed in 30-year-old stands. The visual pattern from the NMDS was confirmed by one-way ANOSIM (Table 2).

**Table 2:** R-values from one-way analysis of similarity (ANOSIM). Comparison of lichen species composition on four trees (standardized by plot numbers) within *Picea abies*, *Pinus contorta*, and *Pinus sylvestris* stands of different age classes. Significant (Bonferroni-corrected *p*-values < 0.05) and meaningful differences (R-values ≥ 0.5) in species composition are given in bold. The single 85 year old *P*. *contorta* stand was not included in the analysis.

		*Picea abies*		*Pinus contorta*		*Pinus sylvestris*		
		15 yr	30 yr	85 yr	15 yr	30 yr	15 yr	30 yr
*Picea abies*	30 yr	**0.454**						
	85 yr	**0.833**	**0.776**					
*Pinus contorta*	15 yr	**0.465**	**0.874**	**0.892**				
	30 yr	**0.463**	**0.886**	**0.799**	**0.438**			
*Pinus sylvestris*	15 yr	**0.625**	**0.990**	**0.909**	0.001	**0.649**		
	30 yr	**0.510**	**0.983**	**0.786**	**0.433**	**0.630**	**0.527**	
	85 yr	**0.894**	**0.915**	**0.587**	**0.920**	**0.912**	**0.997**	**0.963**

In 15-year-old stands, the lichen community of *P*. *abies* stands was influenced by higher branch density. In pine stands, lichen composition of both pine species was influenced by basal area, canopy cover, and diameter at breast height while bark crevice depth was more important in *P*. *sylvestris* stands than in *P*. *contorta* stands (Fig 4a, Table 1). The 30-year-old *P*. *abies* stands were influenced by higher branch density and greater canopy cover, while the lichen assemblages on 30-year-old *P*. *contorta* were influenced by greater canopy cover, basal area, and diameter at breast height (Fig 4b, Table 1). In the 30-year-old *P*. *sylvestris* stands, greater bark crevice depth was the most important factor explaining lichen composition (Fig 4b, Table 1). The species composition of the single 85-year-old *P*. *contorta* stand did not separate from the 85-year-old *P*. *sylvestris* stands (Fig 4c). The lichen species composition of 85-year-old *P*. *sylvestris* stands were influenced by greater basal area, bark crevice depth, and average diameter at breast height, while the lichen composition in 85-year-old *P*. *abies* stands was strongly correlated to branch density and canopy cover (Fig 4c, Table 1).

**Fig 5:**
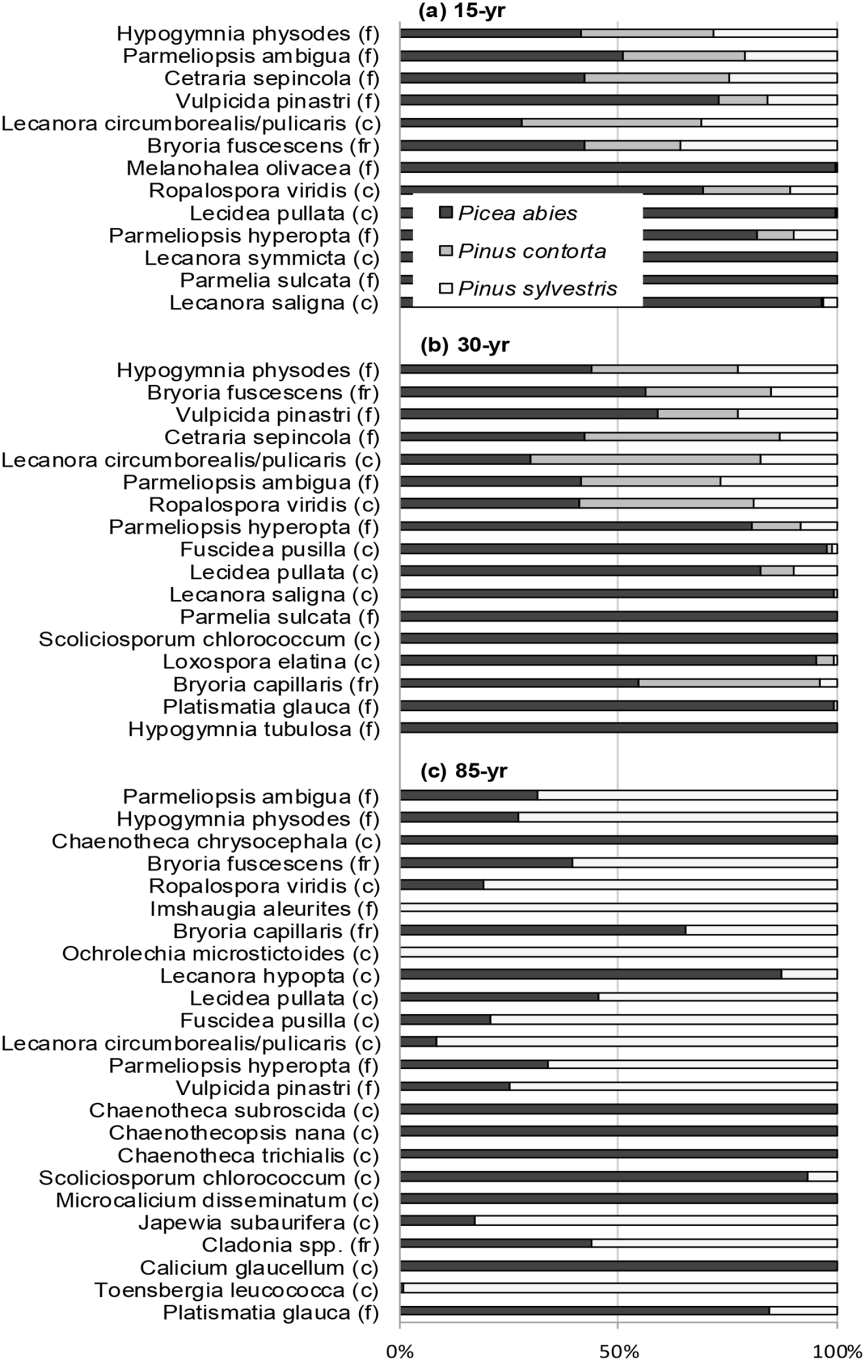
a-c. Similarity percentage analysis (SIMPER) of lichen community dissimilarity between the different tree species. Panels show *Picea abies*, *Pinus contorta*, and *Pinus sylvestris* in (a) 15-year-old, (b) 30-year-old, and (c) 85-year-old forest stands. The single 85-year-old *P*. *contorta* stand was not included in the analysis. Species from the top of the list contributed the most to the average dissimilarity of lichen assemblages in different stand types. Only species that contributed to 90% of the dissimilarity between stand types are presented. Lichen growth form is indicated by the letter in brackets: (c) = crustose, (f) = foliose, and (fr) = fruticose.

The overall multi-group dissimilarity in species abundance was 41% among all 15-year-old stands, 37% for 30-year-old stands, and 55% for 85-year-old *P*. *abies* and *P*. *sylvestris* stands (Fig 5a–5c). Some lichen species were common in all stand types, e.g. *Hypogymnia physodes*, *Parmeliopsis ambigua*, and *Bryoria fuscescens*, but the calicioid lichens (*Calicium glaucellum*, *Chaenotheca chrysocephala*, *C*. *subroscida*, *Chaenothecopsis nana*, and *Microcalicium disseminatum*) were confined to 85-year-old stands of *P*. *abies*. Two species, *Imshaugia aleurites* and *Ochrolechia microstictoides*, were present in 85-year-old *P*. *sylvestris* and *P*. *contorta* stands but absent in 85-year-old *P*. *abies* stands. Crustose lichens progressively became more abundant (higher relative percentage contribution, Fig 5a–5c) throughout the chronosequence, and of the 10 species with highest relative abundance on 85-year-old *P*. *abies*, 8 were crustose lichens. In 85-year-old *P*. *sylvestris*, only half (5) of the species were crustose.

### Lichen cover

**Fig 6:**
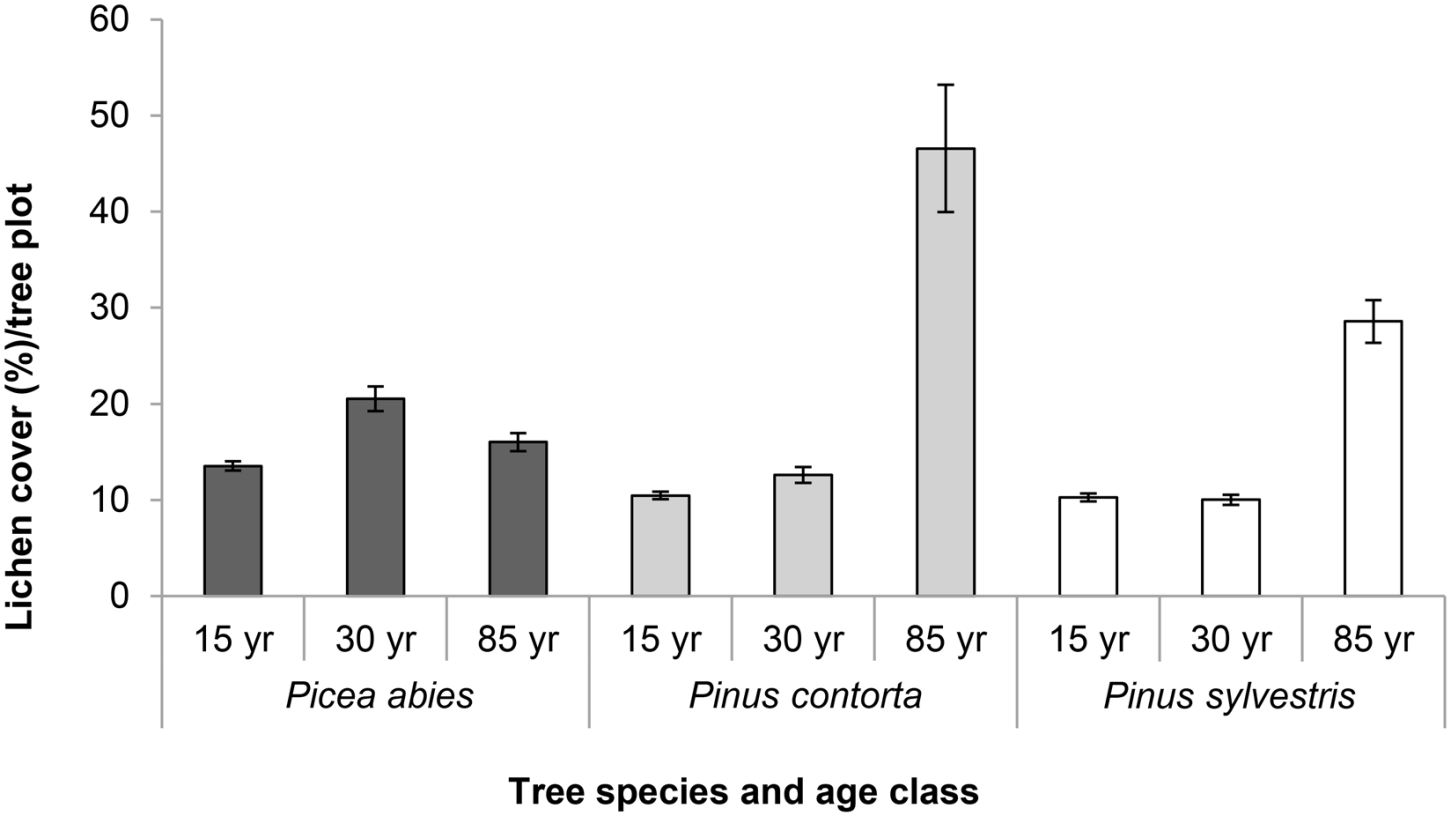
Average lichen cover per tree plot in the different stand types. Error bars represent the standard error.

Lichen cover increased with increasing stand age for both pine species (ANOVA; F _P.contorta df = 1, 94_ = 4.95, P = 0.028, ANOVA; F _P.sylvestris df = 2, 141_ = 62.17, P < 0.001, Fig 6). In *P*. *abies* stands, the average lichen cover decreased between 30-year-old and 85-year-old stands (ANOVA; F _P. abies df = 2, 137_ = 13.52, P < 0.001; Tukey’s pairwise comparison *P*. *abies* 30 yr–*P*.*abies* 85 yr; of means: -0.0445, T = -3.29, P = 0.004). Although the cover of individual lichen species was not recorded, the greater abundance of larger fruticose and foliose lichen species in pine stands implies that these acquire larger cover over time in pine forest stands. This is in contrast to crustose-dominated *P*. *abies* stands with lower overall lichen cover, and this might explain the difference between spruce and pine stands (S2 Table).

## Discussion

### Species richness

Both tree species and stand age had clear effects on lichen species richness. We found the total species numbers to be higher in stands of *P*. *abies* than both *Pinus* species, which corresponds to earlier studies by Marmor et al. [21, 53] and reflects our assumption that lichen species richness would differ between tree species. Other studies focusing on natural forests [28] and the number of target species in managed stands [27] have found little differences in species richness between *P*. *abies* and *P*. *sylvestris*. However, our study is the first to investigate epiphytic lichen species richness on *P*. *contorta* as a non-native tree species. Contrary to our initial expectation, species richness in stands of *P*. *contorta* and *P*. *sylvestris* was of the same magnitude. However, in its natural distribution range, epiphytic lichen species richness has been shown to be lower on *P*. *contorta* trunks than on the moister habitats of Douglas fir (*Pseudotsuga menziesii*) and Engelmann spruce (*Picea engelmannii*) [54], which agrees with the difference in species richness we found between *P*. *contorta* and *P*. *abies* stands.

In accordance with our predictions, stand age was positively associated with lichen species richness, and total species richness increased throughout the chronosequence. Lichen species richness is generally considered to increase with increasing stand age (e.g. [27, 22, 55]), and it has been suggested that boreal lichen communities are additive systems where early colonizers persist and new species are added without replacing the existing species [27, 22]. This also seems to be the case for the different tree species in our study, and most species found in 15-year-old stands were present in 85-year-old stands.

Contrary to our initial hypothesis, stand age was, after correcting for sample-size effects, negatively correlated with species richness in *P*. *abies* stands. Stable or declining species richness numbers with increasing stand age have been reported by others [22, 3]. Declining species richness was found in stands older than 200 years, and this decline was attributed to lower numbers of deciduous trees within these stands [3]. These stands were much older than the stands in our study, and a better comparison is the stable lichen species numbers found in middle-aged stands (40–60 years old) reported by Hilmo et al. [22], which was attributed to low light conditions.

Due to forest management and forest succession, the structure of the forest changes over time [15] and stands of *P*. *abies* often have higher stem densities than *Pinus* spp. at the end of the rotation period. Consequently, more substrate is available for lichen colonization and establishment in older stands of *P*. *abies*, and high species richness might be expected. However, lichen colonization, establishment, and growth might be restricted by environmental factors [17]. In our study, *P*. *abies* had the highest canopy cover and branch density in old (85 years old) stands, which limits the overall light availability in the stand and on the trunk. The lower light availability in *P*. *abies* stands might be suboptimal for many lichen species, and the available substrate might not be fully utilized [56]. In more open older stands of *Pinus* spp. in our study, light was likely not a limiting factor for the colonization, establishment, and growth of epiphytic lichens. This might explain the higher plot-level lichen cover in the oldest 85-year-old *Pinus* stands and the temporal development of species richness of *Pinus* spp. throughout the chronosequence [56].

### Species composition

In accordance with our expectations, the same environmental factors that were important for species richness were also the ones best explaining species composition. Branch density—and canopy cover in the older stands—had a stronger influence on lichen species composition in *P*. *abies* stands than in *Pinus* stands, and this demonstrates the importance of light availability. Likewise, Ódor et al. [17] found light availability to be one of the most important factors explaining stand-level lichen species composition in managed temperate forests. Light-demanding epiphytic lichens are more often found in pine stands than in stands of tree species that provide more shaded conditions [29]. As trees grow larger, habitat availability for epiphytes increases [19]. Structural changes such as coarser bark, increasing diameter, and larger basal area all indicate that more habitat is available in terms of both surface area and surface variability [19, 3]. We found that these factors were important for determining lichen species composition on pines. Bark crevice depth was the factor separating *P*. *sylvestris* from *P*. *contorta*, and the coarser bark of *P*. *sylvestris* probably provides a more diverse habitat for lichens [27, 25]. Large tree diameter and large basal area, which indicate greater habitat availability, were correlated with species assemblages on *Pinus contorta*.

In all tree species, and across all stand ages, lichen communities were dominated by commonly occurring generalist species. Hence, differences in species composition were more related to differences in abundance of these common species than in the occurrence of unique species. However, there were a few species that were specific to stands of a particular tree species or stand age, and the number of unique species differed between pine and spruce.

The most striking difference between pine and spruce was the calicioid species that were present only in 85-year-old spruce stands. Other studies have found at least some of those species (*Calicium glaucellum* and *Chaenotheca chrysocephala*) on pines over 100 years old [53, 28], but in our study this was not the case, probably partly because the pines in our study were too young. Also, stands in the study by Kuusinen [28] were most likely denser and of a mixed spruce-pine character resulting in stands more similar to our spruce stands. Calicioid lichens are often described as associated with old-growth forests, but their association with old-growth forests might depend on geographical context, i.e. one species that is found on one type of substrate in one place can be confined to another substrate in another geographical region [23]. Interestingly, our study indicates that suitable habitat conditions for calicioid lichens, including some red-listed species, can be found in 85-year old managed forests of *P*. *abies*.

In contrast to spruce, not many lichen species were specific to pines. This agrees with findings from native forests in North America, where *P*. *contorta* hosts very few unique species in comparison to *P*. *menziesii* and *P*. *engelmannii* [54]. Furthermore, in Fennoscandia, *P*. *sylvestris* has been reported to have lower numbers of unique species than *P*. *abies* [57]. We observed *Ochrolechia microstictoides* in 85-year-old stands of *P*. *sylvestris* and *P*. *contorta*, but that species was missing from *P*. *abies* stands. *Imshaugia aleurites* was also absent from 85-year-old *P*. *abies* stands but had some occurrences in 30-year-old stands. However, in accordance with other studies (e.g. [58]) *I*. *aleurites* was more common on *P*. *sylvestris* and on *P*. *contorta* than on *P*. *abies*.

Although *P*. *contorta* and *P*. *sylvestris* shared most lichen species, some had higher relative abundance in *P*. *contorta* stands and thereby contributed to differences in species composition in 30-year-old stands. *Cetraria sepincola* and *Lecanora* spp. are considered typical for young forests, but they also grow better with high basal area and thick canopy cover [58]. This corresponds to the structural differences between *Pinus* species in our study and indicates that the higher canopy cover and basal area of *P*. *contorta* provide a more shaded habitat suitable for these species.

Our study showed increasing average lichen cover in stands of both *Pinus* species throughout the chronosequence, while the lichen cover decreased in *P*. *abies* stands between 30 and 85 years old. This is probably connected to the fact that larger-sized foliose and fruticose lichens are more abundant and can expand their cover on pines due to favorable light conditions. The smaller-sized crustose lichen that can tolerate low light conditions [58] instead became dominating on older spruce trees.

It is well-established that tree species that resemble each other in terms of the structural environment that they give rise to often support similar communities of associated species (e.g. [12]), thus our finding that the lichen community of the non-native *P*. *contorta* was more similar to the native *P*. *sylvestris* than the phylogenetically more distant native spruce *P*. *abies* was not surprising. *Picea abies* had a distinct lichen composition and higher species richness in all age classes, while the species richness of *P*. *contorta* was comparable to *P*. *sylvestris*. The environmental factors of mature pine stands appear similar in terms of canopy cover, branch density, and stem density. However, the structure of the bark differs between the species, causing a differentiation in lichen species composition in 30-year-old stands. It is possible that bark structure might also cause species differentiation in mature stands because the bark of *P*. *contorta* is more similar to *P*. *abies* in the upper part (rather smooth and not exfoliating like *P*. *sylvestris*, personal observation) but of intermediate coarseness in the lower part of the tree (Table 1). We know very little about the vertical distribution of epiphytic lichens in mature managed boreal forests, and even less about the vertical distribution of lichens on non-native tree species. This was not possible to assess in this study, but other studies have shown that the lichen species composition in mature forest stands can vary with tree height and that the upper canopy of old trees can host distinctive epiphyte assemblages [59, 60].

Although studies of mature stands are lacking, younger stands of introduced *P*. *contorta* are not “biological deserts” from the perspective of epiphytic species (this study), understory species [61, 62, 63], or epigaeic beetles [62]. The higher growth rate of the non-native *P*. *contorta* does not affect lichen species richness in comparison to native *P*. *sylvestris*, and the main difference in species richness and composition is between *P*. *abies* and both of the *Pinus* species. How the introduction of *P*. *contorta* will affect epiphytic lichens in the future remains to be studied, but it is important to keep monitoring the epiphytic flora in mature stands to assess the implications of the full rotation cycle of this tree species on native lichen biodiversity in Sweden.

## Supporting Information

S1 Dataset Plotlevel occurrences of lichen species. (XLSX)

S2 Dataset Dataset used for GLMM. (XLSX)

S3 Dataset Stand level data. (XLSX)

S4 Dataset Lichen cover data. (XLSX)

S1 Fig A schematic illustration of the tree plot used for lichen inventory. (PDF)

S2 Fig The species richness variation within and between stands. (PDF)

S3 Fig Relative variable importance (RVI) of stand-level explanatory variables. (PDF)

S1 Model Model statistics from the GLMMs. (PDF)

S1 Table List of the 57 lichen species recorded. (PDF)

S2 Table Contribution (%) of different lichen growth forms (crustose, foliose, and fruticose) in the different stand types and for all tree species and stand ages pooled (All). (PDF)
