# Supplementary material for: A Pine Is a Pine and a Spruce Is a Spruce – The Effect of Tree Species and Stand Age on Epiphytic Lichen Communities
Source: PLoS One. 2016 Jan 22;11(1):e0147004. doi: 10.1371/journal.pone.0147004 (PMC4723141; doi:10.1371/journal.pone.0147004)
Supplement: S1 Fig — (PDF) [file pone.0147004.s005.pdf]

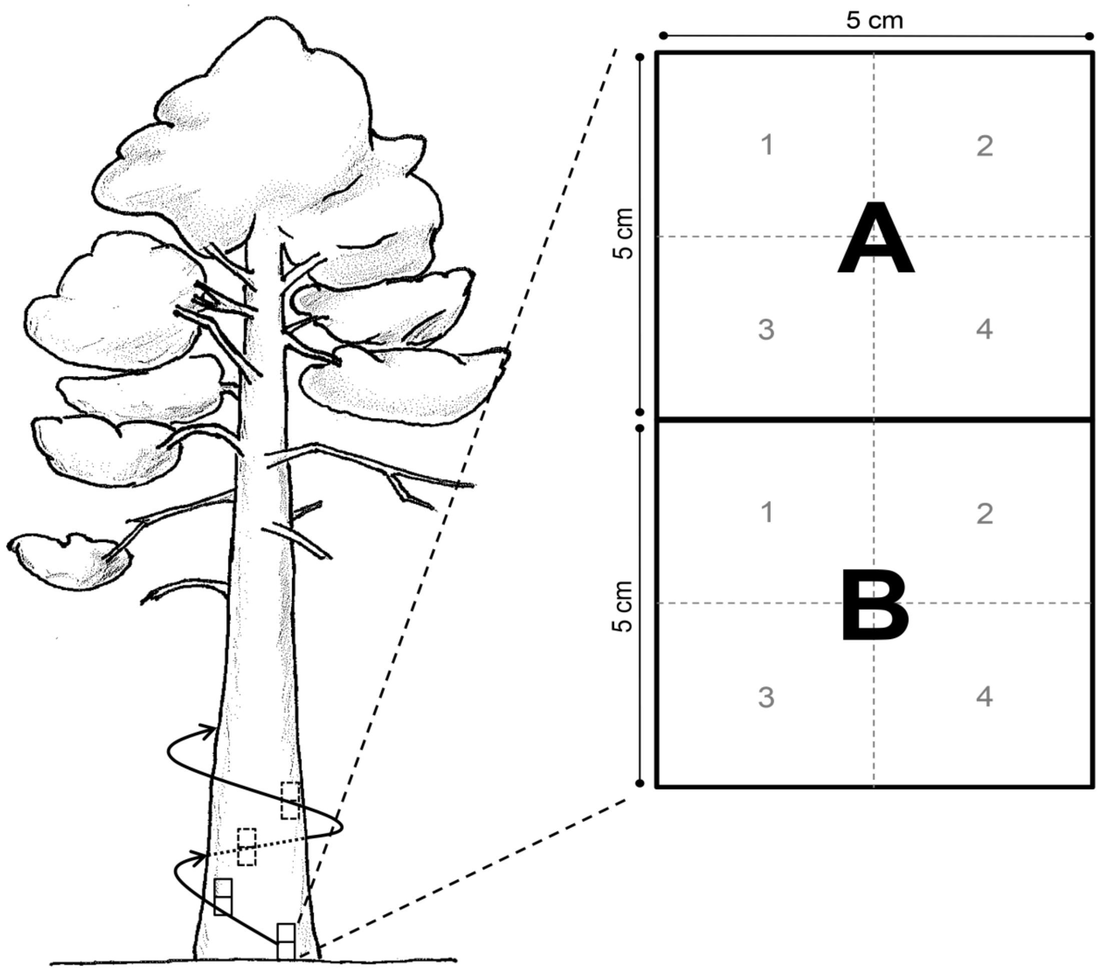

S1 Figure. A schematic illustration of the tree plot used for lichen inventory. Two adjacent plots (A and B) were used, each divided into four subplots for abundance measure. The plot was rotated upwards around the tree with one cardinal direction (N, E, S, W) between each consecutive plot. The plot was first placed at ground level (0 cm), and then 25, 50, 75 and so on. From 200 cm above ground, the plot distance was extended to 50 cm (i.e., 200, 250, 300 and so on).
