# Supplementary material for: A Pine Is a Pine and a Spruce Is a Spruce – The Effect of Tree Species and Stand Age on Epiphytic Lichen Communities
Source: PLoS One. 2016 Jan 22;11(1):e0147004. doi: 10.1371/journal.pone.0147004 (PMC4723141; doi:10.1371/journal.pone.0147004)
Supplement: S2 Fig — (PDF) [file pone.0147004.s006.pdf]

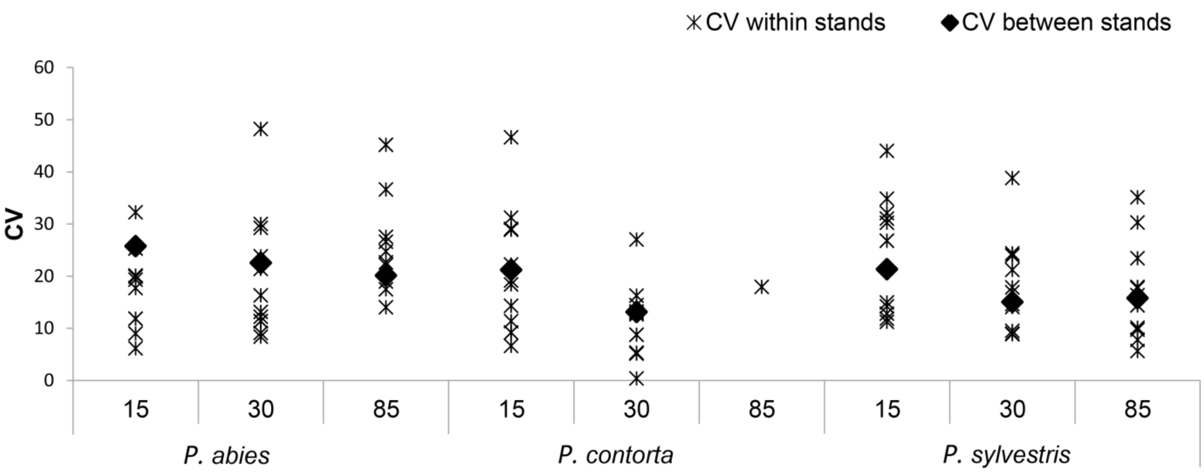

S2 Fig. The species richness variation within and between stands. The coefficient of variation (CV) within each stand (cross) and between stands within each stand type (filled diamond).
