# Supplementary material for: A Pine Is a Pine and a Spruce Is a Spruce – The Effect of Tree Species and Stand Age on Epiphytic Lichen Communities
Source: PLoS One. 2016 Jan 22;11(1):e0147004. doi: 10.1371/journal.pone.0147004 (PMC4723141; doi:10.1371/journal.pone.0147004)
Supplement: S3 Fig — (PDF) [file pone.0147004.s007.pdf]

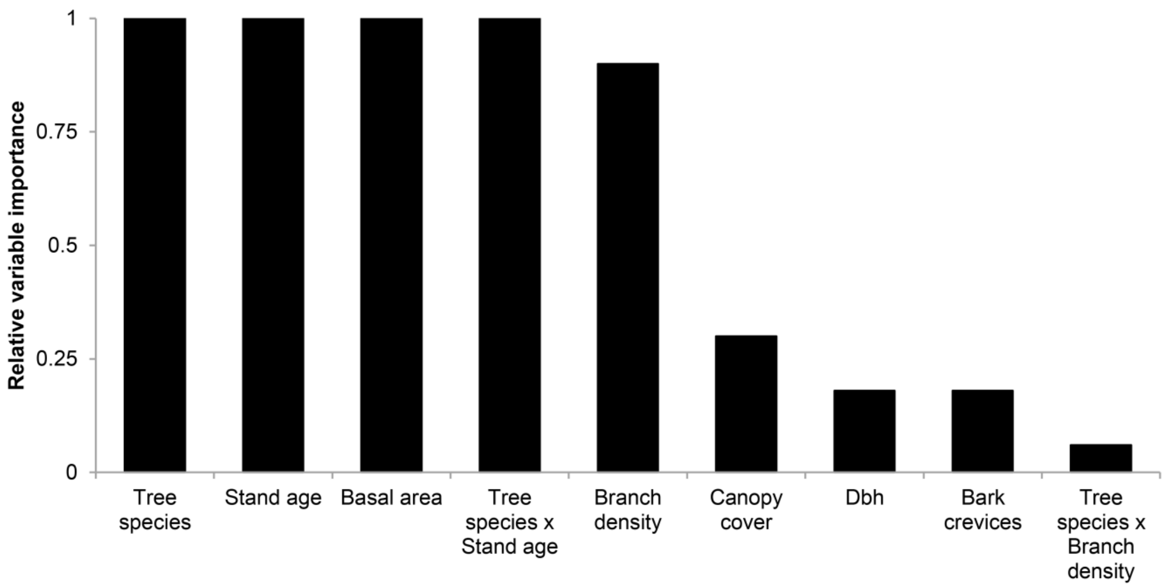

S3 Fig. Relative variable importance (RVI) of stand-level explanatory variables for total lichen species richness in *Picea abies*, *Pinus contorta,* and *Pinus sylvestris* stands of three different age classes.
