# Supplementary material for: A Pine Is a Pine and a Spruce Is a Spruce – The Effect of Tree Species and Stand Age on Epiphytic Lichen Communities
Source: PLoS One. 2016 Jan 22;11(1):e0147004. doi: 10.1371/journal.pone.0147004 (PMC4723141; doi:10.1371/journal.pone.0147004)
Supplement: S1 Model — (PDF) [file pone.0147004.s008.pdf]

Supplementary material: Models used in multimodel inference

Contains: Stand-level GLMM:s – output from multimodel inference in R

Stand-level GLMM:s – output from multimodel inference in R

Explanatory variables:

Treesp = Tree species (*Pinus contorta* as reference)
ageT = Stand age
basalarea = Tree basal area
dbh = Diameter at breast height
cc.prop = Canopy cover
barkcrev = Bark crevice depth
bra.den = Branch density

Formulas used:

```
modell = lmer(<response> ~ treesp + ageT + basalarea + dbh + cc.prop + barkcrev + bra.den +
treesp:ageT + treesp:basalarea + treesp:dbh + treesp:cc.prop + treesp:barkcrev + treesp:bra.den +
(1|site), offset = log (number of plots), family = poisson)
```

Response variable: Total lichen species richness

```
Component models:
        df  logLik   AICc Delta Weight
12458    9 -160.89 340.26  0.00   0.32
124568  10 -160.44 341.47  1.21   0.18
123458  10 -160.87 342.32  2.07   0.11
124578  10 -160.88 342.34  2.08   0.11
1248     8 -163.14 342.66  2.40   0.10
1245678 11 -160.40 343.51  3.26   0.06
1234568 11 -160.43 343.57  3.31   0.06
124589  11 -160.52 343.75  3.50   0.06

Term codes:
          treesp           z.ageT       z.barkcrev      z.basalarea 
               1                2                3                4 
       z.bra.den       z.cc.prop            z.dbh    treesp:z.ageT 
               5                6                7                8 
treesp:z.bra.den 
               9 

Model-averaged coefficients:
                  Estimate Std. Error z value Pr(>|z|)    
(Intercept)       -1.21733    0.05417  22.474  < 2e-16 ***
treespg            0.86323    0.06810  12.676  < 2e-16 ***
treespt            0.04951    0.06514   0.760 0.447220    
z.ageT             0.57428    0.16423   3.497 0.000471 ***
z.basalarea       -0.17288    0.06157   2.808 0.004989 ** 
z.bra.den         -0.07641    0.05119   1.493 0.135491    
treespg:z.ageT    -0.91129    0.16221   5.618  < 2e-16 ***
treespt:z.ageT    -0.23429    0.15909   1.473 0.140840    
z.cc.prop         -0.05182    0.05457   0.950 0.342331    
z.barkcrev         0.01235    0.06622   0.187 0.852009    
z.dbh             -0.01457    0.07331   0.199 0.842512    
treespg:z.bra.den -0.06891    0.13312   0.518 0.604700    
treespt:z.bra.den -0.12555    0.15125   0.830 0.406495    
---
Signif. codes:  0 ‘***’ 0.001 ‘**’ 0.01 ‘*’ 0.05 ‘.’ 0.1 ‘ ’ 1

Full model-averaged coefficients (with shrinkage):
 (Intercept)    treespg    treespt     z.ageT z.basalarea  z.bra.den 
  -1.2173305  0.8632305  0.0495118  0.5742816  -0.1728789 -0.0690387 
```

```
 treespg:z.ageT treespt:z.ageT  z.cc.prop z.barkcrev      z.dbh
     -0.9112937     -0.2342901 -0.0155098  0.0021685 -0.0025659
 treespg:z.bra.den treespt:z.bra.den
       -0.0038511        -0.0070164

Relative variable importance:
          treesp           z.ageT      z.basalarea    treesp:z.ageT
            1.00             1.00             1.00             1.00
       z.bra.den        z.cc.prop            z.dbh       z.barkcrev
            0.90             0.30             0.18             0.18
treesp:z.bra.den
            0.06
> confint(avgmod.95p)
                        2.5 %      97.5 %
(Intercept)       -1.32349241 -1.11116867
treespg            0.72976154  0.99669951
treespt           -0.07816437  0.17718793
z.ageT             0.25240440  0.89615873
z.basalarea       -0.29355851 -0.05219923
z.bra.den         -0.17673792  0.02391325
treespg:z.ageT    -1.22922185 -0.59336550
treespt:z.ageT    -0.54610451  0.07752422
z.cc.prop         -0.15878291  0.05514053
z.barkcrev        -0.11744004  0.14214848
z.dbh             -0.15825112  0.12912029
treespg:z.bra.den -0.32982763  0.19200443
treespt:z.bra.den -0.42200402  0.17089951
```
