# Supplementary material for: A Pine Is a Pine and a Spruce Is a Spruce – The Effect of Tree Species and Stand Age on Epiphytic Lichen Communities
Source: PLoS One. 2016 Jan 22;11(1):e0147004. doi: 10.1371/journal.pone.0147004 (PMC4723141; doi:10.1371/journal.pone.0147004)
Supplement: S1 Table — (PDF) [file pone.0147004.s009.pdf]

S1 Table. List of the 57 lichen species recorded.

Nubers indicating occurrence (%) of each lichen species in the subplots of the different stand types.

		Picea abies			*Pinus contorta*			*Pinus sylvestris*		
**Lichen species**	**Growth form**	**15 yrs**	**30 yrs**	**85 yrs**	**15 yrs**	**30 yrs**	**85 yrs**	**15 yrs**	**30 yrs**	**85 yrs**
Alectoria sarmentosa	Fruticose	-	0.04	0.48	-	-	-	-	-	0.14
Amandinea punctata	Crustose	0.30	0.30	0.55	-	0.08	-	-	0.22	1.00
Biatora albohyalina	Crustose	-	-	-	-	-	4.75	-	-	0.14
Biatora meiocarpa	Crustose	-	0.02	0.04	-	-	-	-	-	-
Bryoria capillaris	Fruticose	0.35	1.89	7.24	0.25	1.35	0.07	0.26	0.13	1.44
Bryoria fremontii	Fruticose	-	-	0.02	-	-	-	-	-	0.01
Bryoria fuscescens	Fruticose	11.24	34.61	28.04	5.36	17.35	38.15	8.22	9.00	3.56
Calicium glaucellum	Crustose	-	-	1.42	-	-	-	-	-	-
Calicium viride	Crustose	-	-	0.28	-	-	-	-	-	0.01
Cetraria sepincola	Foliose	15.54	16.16	1.37	14.07	16.86	1.76	9.68	4.73	6.17
Chaenotheca chrysocephala	Crustose	-	-	25.02	-	-	-	-	-	5.23
Chaenotheca subroscida	Crustose	-	-	3.00	-	-	-	-	-	-
Chaenotheca trichialis	Crustose	-	-	2.50	-	-	-	-	-	0.18
Chaenothecopsis consociata	Crustose	-	-	0.02	-	-	-	-	-	-
Chaenothecopsis nana	Crustose	-	-	2.81	-	-	-	-	-	-
Cladonia spp. (phyllocladia)	Fruticose	2.56	1.76	1.26	0.05	0.20	3.13	0.23	0.59	-
Fuscidea pusilla	Crustose	1.39	9.03	1.23	-	0.11	0.33	0.03	0.13	5.44
Hypocenomyce scalaris	Crustose	-	-	-	-	-	-	-	-	-
Hypogymnia physodes	Foliose	58.85	76.19	17.33	41.78	57.20	40.76	37.82	37.89	59.43
Hypogymnia tubulosa	Foliose	0.17	2.36	0.09	-	-	-	-	-	39.77
Imshaugia aleurites	Foliose	-	0.13	-	-	0.05	2.93	-	0.05	0.24
Japewia subaurifera	Crustose	-	0.06	0.37	-	0.03	2.21	-	0.03	-
Japewia tornoënsis	Crustose	-	0.06	0.05	-	-	-	-	-	-
Lecanora circumborealis/pulicaris	Crustose	6.08	10.74	0.44	9.57	19.39	0.13	6.57	6.21	-
Lecanora hypopta	Crustose	0.52	1.45	5.81	-	0.19	-	-	0.03	0.04
Lecanora saligna	Crustose	3.47	7.48	0.30	0.02	0.05	0.26	0.12	-	0.17
Lecanora symmicta	Crustose	3.34	2.08	0.18	-	0.04	-	-	-	1.49
Lecidea erythrophaea	Crustose	-	-	0.02	-	-	0.65	-	-	-
Lecidea nylanderi	Crustose	0.04	0.45	0.60	-	-	-	-	-	-
Lecidea pullata	Crustose	4.95	7.82	5.10	0.02	0.66	6.38	-	0.99	4.76
Lecidea turgidula	Crustose	-	-	0.02	-	-	-	-	-	-
Lepraria jackii	Crustose	0.04	0.11	1.14	-	-	-	-	-	-
Loxospora elatina	Crustose	0.82	3.15	0.94	-	0.12	0.07	-	0.02	-
Melanohalea olivacea	Foliose	9.20	1.74	0.11	-	-	-	0.03	-	0.39
Micarea denigrata/nowakii	Crustose	-	0.02	-	-	-	-	-	-	47.63
Micarea micrococca	Crustose	-	0.15	0.02	-	-	-	-	-	-
Micarea prasina	Crustose	0.09	0.19	0.07	-	-	0.13	-	-	-
Microcalicium disseminatum	Crustose	-	-	1.58	-	-	-	-	-	-
Mycoblastus affinis/M. alpinus	Crustose	0.04	0.17	1.33	-	-	0.20	-	-	0.14
Mycoblastus sanguinarius	Crustose	-	0.04	0.62	-	-	1.04	-	-	0.06
Ochrolechia androgyna	Crustose	0.13	0.06	0.30	-	-	-	-	-	-
Ochrolechia brodoi	Crustose	-	0.04	0.34	-	0.44	1.43	-	0.03	0.28
Ochrolechia microstictoides	Crustose	-	-	-	-	-	23.05	-	-	0.73
Parmelia sulcata	Foliose	3.39	5.31	0.83	-	-	-	-	-	-
Parmeliopsis ambigua	Foliose	25.04	37.59	28.04	14.05	29.36	76.50	10.01	24.84	4.56
Parmeliopsis hyperopta	Foliose	4.30	10.67	3.53	0.44	1.43	4.17	0.50	1.04	6.01
Pertusaria borealis/P. pupillaris	Crustose	0.09	0.15	-	-	-	-	-	-	-
Platismatia glauca	Foliose	0.56	2.59	1.53	-	0.01	0.78	-	-	-
Ramboldia cinnabarina	Crustose	-	-	-	-	-	0.98	-	-	0.15
Rinodina septentrionalis	Crustose	0.04	0.02	-	-	-	-	-	-	-
Ropalospora viridis	Crustose	6.42	14.69	3.60	1.89	13.62	25.33	0.94	6.58	4.05
Scoliciosporum chlorococcum	Crustose	1.82	3.70	2.11	-	-	0.46	0.03	-	0.39
Toensbergia leucococca	Crustose	0.22	0.66	0.02	-	0.15	0.59	-	0.35	1.63
Tuckermannopsis chlorophylla	Foliose	0.30	2.34	0.64	-	-	-	-	-	0.04
Usnea spp.	Fruticose	0.04	0.11	-	-	0.03	0.07	-	0.02	-
Violella fucata	Crustose	-	-	0.04	-	-	-	-	-	-
Vulpicida pinastri	Foliose	19.66	27.64	1.49	3.05	8.51	10.22	4.17	10.59	0.68
