# Supplementary material for: A Pine Is a Pine and a Spruce Is a Spruce – The Effect of Tree Species and Stand Age on Epiphytic Lichen Communities
Source: PLoS One. 2016 Jan 22;11(1):e0147004. doi: 10.1371/journal.pone.0147004 (PMC4723141; doi:10.1371/journal.pone.0147004)
Supplement: S2 Table — (PDF) [file pone.0147004.s010.pdf]

S2 Table. Contribution (%) of different lichen growth forms (crustose, foliose and fruticose) in the different stand types and for all tree species and stand ages pooled (All).

**Tree species**	**Age class**	**Growth form (%)**		
		Crustose	Foliose	Fruticose
Picea abies	15	16.5	75.7	7.8
	30	22.1	64.4	13.5
	85	40.2	35.7	24.1
Pinus contorta	15	12.7	81	6.2
	30	20.9	67.8	11.3
	85	27.6	55.6	16.8
Pinus sylvestris	15	9.8	79.1	11.1
	30	14.1	76.5	9.4
	85	20.6	58.5	21
All		22	62.9	15
